# The Potential Role of 8-Oxoguanine DNA Glycosylase-Driven DNA Base Excision Repair in Exercise-Induced Asthma

**DOI:** 10.1155/2016/3762561

**Published:** 2016-07-25

**Authors:** KarryAnne K. Belanger, Bill T. Ameredes, Istvan Boldogh, Leopoldo Aguilera-Aguirre

**Affiliations:** ^1^Department of Internal Medicine, University of Texas Medical Branch, Galveston, TX 77555, USA; ^2^Department of Biochemistry and Molecular Biology, University of Texas Medical Branch, Galveston, TX 77555, USA; ^3^Sealy Center for Molecular Medicine, School of Medicine, University of Texas Medical Branch, Galveston, TX 77555, USA; ^4^Sealy Center for Environmental Health and Medicine, School of Medicine, University of Texas Medical Branch, Galveston, TX 77555, USA; ^5^Department of Microbiology and Immunology, University of Texas Medical Branch, Galveston, TX 77555, USA

## Abstract

Asthma is characterized by reversible airway narrowing, shortness of breath, wheezing, coughing, and other symptoms driven by chronic inflammatory processes, commonly triggered by allergens. In 90% of asthmatics, most of these symptoms can also be triggered by intense physical activities and severely exacerbated by environmental factors. This condition is known as exercise-induced asthma (EIA). Current theories explaining EIA pathogenesis involve osmotic and/or thermal alterations in the airways caused by changes in respiratory airflow during exercise. These changes, along with existing airway inflammatory conditions, are associated with increased cellular levels of reactive oxygen species (ROS) affecting important biomolecules including DNA, although the underlying molecular mechanisms have not been completely elucidated. One of the most abundant oxidative DNA lesions is 8-oxoguanine (8-oxoG), which is repaired by 8-oxoguanine DNA glycosylase 1 (OGG1) during the base excision repair (BER) pathway. Whole-genome expression analyses suggest a cellular response to OGG1-BER, involving genes that may have a role in the pathophysiology of EIA leading to mast cell degranulation, airway hyperresponsiveness, and bronchoconstriction. Accordingly, this review discusses a potential new hypothesis in which OGG1-BER-induced gene expression is associated with EIA symptoms.

## 1. Introduction

Globally, more than 300 million people suffer from varying severity of asthma and the annual global death rate associated with it exceeds 250,000 [1]. In the United States, the chronic lower respiratory diseases are the third leading cause of death and more than 30 million people including children and adults have been diagnosed with asthma [2]. Exercise-induced asthma (EIA) is a condition involving acute bronchial narrowing (bronchoconstriction) and other asthma-related symptoms triggered by strenuous physical activity [3]. In persons with EIA, bronchial narrowing typically occurs within 5 to 10 minutes after exercise and normally ceases within 30 and 60 minutes and thereafter [3]. The direct impact of bronchoconstriction in pulmonary function is reflected by a decrease (≥10%) in the forced expiratory volume in 1 second (FEV1) [4]. Although the precise prevalence of EIA is not known, some studies suggest that more than 10% of the general population is affected and 90% of asthmatics also show EIA symptoms [3, 5]. This is an important fact to be considered for asthmatics when they engage in sports and conditioning activities.

The etiology of EIA is not yet fully known, but the major theories explaining the characteristic bronchoconstriction after exercise are the osmotic theory and the thermal theory. The first theory proposes that increased ventilation during exercise results in loss of water from the airway cells causing an osmotic imbalance and ultimately stimulating the release of proinflammatory mediators [6–8]. The second theory proposes that a decrease in airway temperature produced by changes in the respiratory air flow during physical activities results in reduction in airway vascular tone and the consequent rebound vasodilation, leading to hyperemia, edema, and airway obstruction [8]. Both mechanisms (osmotic and thermal) are considered stressors that can potentially increase cellular reactive oxygen species (ROS) [9, 10] and trigger various inflammatory responses including mast cell degranulation, release of histamine, and generation of lipoperoxidation products such as leukotriene D4 (LTD4), leading to contractions in airway smooth muscles and ultimately bronchoconstriction [11, 12].

The susceptibility of individuals for EIA depends on their genetic background and the environment-modulated epigenetic changes [13, 14]. Initiation, immunopathogenesis, and pathophysiology of EIA result from a highly complex interplay among dysregulated airway epithelial, smooth muscle, and mast cells, and are manifested via a multitude of mediators leading to airway narrowing [15]. These events have also been linked to increased levels of ROS and the use of antioxidants has been shown to reduce exercise-induced bronchoconstriction [16, 17]. In addition, system biology approaches suggested that the repair product of oxidatively damaged DNA by OGG1 induces gene expression associated with airway inflammation, asthma, and EIA related symptoms [18–21]. Thus, this review proposes and discusses a potential role of OGG1-BER induced gene expression in the pathophysiology of EIA.

## 2. Oxidative Stress and EIA

Oxidative stress is characterized by an imbalance between the production of ROS and the antioxidant defenses, in which the ability of the antioxidant defense to neutralize ROS is overwhelmed. Importantly, ROS are also signaling species that promote airway inflammation and are etiologically linked to exacerbation of asthma by stimulating bronchial hyperreactivity, mast cell degranulation (e.g., release of histamine), generation of mucus, and induction of proinflammatory gene expression [23–25]. As a part of the ROS-driven stress responses and processes within the airway, two of the important redox reactions that can occur and may play a role in EIA are lipid peroxidation and DNA oxidation.

### 2.1. Lipid Peroxidation

The products of the isoprostane pathway are considered excellent biomarkers of oxidative stress in the airways [26]. A good example of these is 8-epi-prostaglandin F2 alpha (8-epi-PGF2 alpha), which has also been shown to cause contraction of smooth muscle by triggering the thromboxane A2 receptor (TBXA2R) [25, 27], and has been shown to have a role in asthma and EIA [28, 29]. Another important lipid peroxidation pathway associated with EIA is the 5-lipoxygenase (5-LO), which transforms essential fatty acids into leukotrienes. This pathway is the major source of potent proinflammatory leukotrienes (LT), such as LTB4, which is an activator and chemoattractant for leukocytes, and is implicated in several inflammatory diseases [30]. Other leukotrienes, such as LTC4 and LTD4, are potent contracting agents of smooth muscle in airways and can induce epithelial mucus secretion [31]. It has been shown that, in EIA, the ratio between prostaglandin E2 (PGE2) and cysteinyl leukotrienes (CysLTs) becomes imbalanced, favoring the latter. This may be important in EIA because PGE2 normally inhibits mast cell degranulation and promotes relaxation of the airway [32]. In support of this concept, it has been shown that elevated CysLTs contribute to exercise-induced bronchoconstriction (EIB), a hallmark of EIA [33, 34]. Leukotriene receptor antagonists such as montelukast have been successfully used to decrease airway responsiveness to various stimuli [35], supporting that lipid peroxidation is an important factor in asthma, and could be potentially important in EIA, as well.

### 2.2. DNA Oxidation

ROS induce oxidative modifications to DNA, including single and double strand breaks, preferentially affecting guanine due to its lowest redox potential among other DNA bases [36–38]. Mechanistic studies show that when ^•^OH interacts with guanine, it results in a reducing neutral radical that reacts with molecular oxygen (O_2_) and, via electron transfer, forms 7,8-dihydro-8-oxoguanine (8-oxoG) [39–41]. To prevent accumulation of oxidized DNA base lesions and their mutagenic effects, eukaryotic cells express DNA repair proteins that are analogous to those from* E. coli* (e.g., formamidopyrimidine DNA glycosylase: MutM; endonuclease VIII-like DNA glycosylase: Nei; 8-oxo-7,8-dihydrodeoxyguanosine triphosphate GTPase) [42]. OGG1 is an eukaryotic MutM homolog that excises 8-oxoG and its open-ring form FapyG from the 8-oxoG/FapyG:C mispairing [43]. Among oxidized DNA base lesions, 8-oxoG is a biomarker of oxidative damage to DNA [44]. The level of genomic 8-oxoG correlates well with the dose and length of exposure, chemical composition, and physical nature of the inhaled agents/oxidants [45–47]. Furthermore, under chronic inflammatory conditions 8-oxoG is one of the most frequent forms of DNA base damage in the genome [48–53]. Recently, it has been proposed that OGG1-driven DNA base excision repair has a role in asthma pathogenesis [21]. In addition, studies using lungs exposed to the BER product of OGG1 (8-oxoG) revealed a role in various biological processes including innate immune responses and inflammation [18]. Thus, it seems plausible that cellular 8-oxoG levels could be associated with EIA.

## 3. OGG1-Driven Proinflammatory Signaling through Small GTPases 

Unexpectedly,* Ogg1* ^−/−^ null mice did not show a marked phenotype despite the increased levels of the mutagenic 8-oxoG in their genomic DNA. More strikingly,* Ogg1* deficiency in mice resulted in decreased inflammatory responses as shown by decreased accumulation of neutrophils, eosinophils and levels of Th1/Th2 cytokines [54–56]. These findings suggested that the absence of 8-oxoG and/or OGG1 is a missing component of proinflammatory signaling in the* Ogg1* ^−/−^ null mice, implicating the importance of OGG1-BER in the inflammatory process.

During OGG1-BER, the 8-oxoG base is excised from DNA and released to the cytoplasm where it complexes with a cytoplasmic OGG1. The conformational changes in the OGG1 molecule allow it to interact with, and activate, RAS family small GTPases, including Kirsten (K)-RAS, neuroblastoma RAS viral oncogene homolog (N)-RAS, and Harvey (H)-RAS [57, 58]. In addition, the RAS homology GTPases RHO and RHO family of GTPases (RAC1, RAC2, and RAC3) can be activated by 8-oxoG-bound OGG1 [59–62]. Thus, the complex functions as a guanine nucleotide exchange factor (GEF) in a manner similar to that of other RAS and RAS homology protein-activating factors [57, 58]. The biological significance of small GTPase activations by OGG1-BER is not fully understood at this time. However, those observations also suggested that increased 8-oxoG levels in DNA or in body fluids observed in asthmatics are not only biomarkers of environmental exposures or inflammatory processes, but they may also serve as a second messenger [63]. In fact, it has been shown that OGG1-BER–mediated activation of GTPases (K-RAS, RHOA, and RAC1) plays a role in the innate allergic inflammatory response [18, 64], maintenance of chronic inflammation [21, 64], and airway remodeling [20, 62]. Therefore, we postulated that OGG1-BER-induced cell signaling is also likely to play important roles in the pathogenesis and progression of EIA.

## 4. Gene Expression Associated with Exercise-Induced Bronchoconstriction

In general, asthma is a multifactorial disease that involves complex cell signaling cascades leading to activation and release of multiple proinflammatory mediators, including cytokines/chemokines, histamine, proteases, and heparin. Despite decades of asthma research, our knowledge about the intricate signaling networks and the fine regulation of gene expression associated with this airway disease remains limited. In the particular case of EIA, there is also limited information on the regulation of global gene expression involved in its pathogenesis. Thus, to gain insight into the gene expression underpinning EIA, it is necessary to compare and integrate available gene expression data with the current next-generation whole-genome RNA-Seq data.

**Figure 1:**
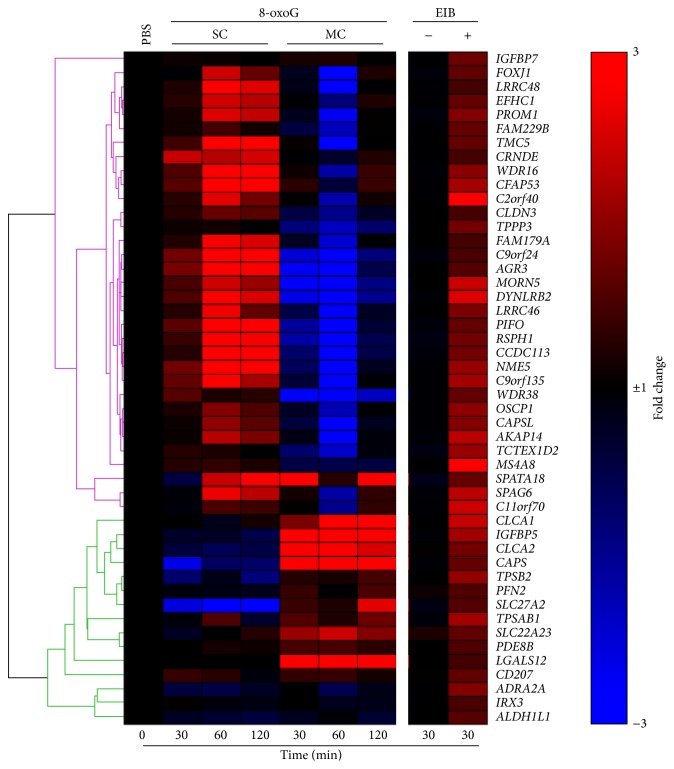
Comparative gene expression between 8-oxoG challenged- and intensive exercise-exposed airways. The clustered heat map was constructed using the list of the most upregulated genes in airway epithelial cells from EIB positive (EIB^+^) individuals after 30 minutes of intensive exercise, as described in Hallstrand et al. [22]. This list of genes was compared to a list of the same genes from lungs challenged once (SC) or multiple times (MC). Top ranked gene list was obtained using Gene Expression Omnibus (GEO)'s built-in application GEO2R (http://www.ncbi.nlm.nih.gov/geo/geo2r/) and Hallstrand et al. GEO deposited dataset (GSE13785). Hierarchically clustered heat map was generated using GENE-E (Broad Institute, http://www.broadinstitute.org/).

We have recently shown that OGG1-BER signaling induces gene expression associated with typical asthma symptoms [19, 20]. Thus, to elucidate the possible role of OGG1-BER signaling in EIA, we compared our RNA-Seq whole-genome gene expression datasets from mouse lungs exposed to 8-oxoG after single challenge (SC, GEO accession number: GSE61095) or multiple challenges (MC, GEO accession number: GSE65031), to the 48 top-ranked upregulated (≥1.5-fold) genes from individuals with exercise-induced bronchoconstriction characteristics (EIB^+^) and those without it (EIB^−^), as a control, after intensive exercise (Table 1) published by Hallstrand et al. (GEO accession number: GSE13785) [22]. Interestingly, most of the top 48 upregulated genes in the EIB^+^ group were also upregulated in the SC group of 8-oxoG challenged lungs (Figure 1, upper cluster). On the other hand, fewer genes were upregulated in the MC group (Figure 1, lower cluster). These observations suggest a link between early gene expression induced in SC group by OGG1-BER cell signaling and the gene expression observed after strenuous exercise in the EIB^+^ group. Interestingly, the few genes induced by repeated stimulation (MC) of OGG1-BER signaling showed association with genes upregulated after exercise in EIB^+^ subjects. Taken together, gene expression profiles induced by both SC and MC might have a role at different stages in the pathogenesis of EIA.

## 5. Mast Cell Degranulation 

Mast cells are one of the most important effector cells involved in elicitation of allergic responses [65]. Antigenic activation of mast cells via the high-affinity receptor for IgE (FcɛRI) mediates exocytosis of cytoplasmic granules containing preformed mediators, secretion of lipid-derived factors, and de novo synthesis of cytokines, chemokines, and growth factors [65–68]. In addition to FcɛRI-mediated signals, exposure to a variety of stimuli including pathogen-associated molecules can lead to the release of mast cell mediators [69].

During allergic and other inflammatory reactions, mast cells are exposed to an oxidative microenvironment milieu made up of ROS produced by various cell types in the surrounding peripheral tissues, as a consequence of their effector function [70]. Accordingly, various studies have shown the relevance of ROS/oxidative stress in mast cell activation [71–73]. For example, we have previously reported that it is not the allergenic proteins in pollen grains, subpollen particles, but ROS (superoxide anion, O_2_^−^) generated by their intrinsic NAD(P)H oxidases that are the primary cause of mast cell degranulation in airways and conjunctiva, during allergen/antigen-induced allergic responses [74–76].

Our studies also showed ROS-enhanced secretion of histamine and serotonin from mast cells, independently from FcɛRI-generated stimuli [77]. The release of biogenic amines in those cells was associated with inhibition of H^+^-ATPase activity within secretory granules, activation of PKC-*δ*, and microtubule-dependent motility and was independent from intracellular free Ca^2+^ levels. We also observed that IgE-mediated mast cell degranulation and antigen-triggered *β*-hexosaminidase release was decreased by ROS, while ROS were synergistic with antigen-induced IL-4 production in sensitized cells. Taken together, these data suggest that ROS and probably its downstream effect of cell signaling through OGG1-BER can act independently from antigen, augmenting the release of biogenic amines [77], and the initiation of antigen-independent effects.

**Figure 2:**
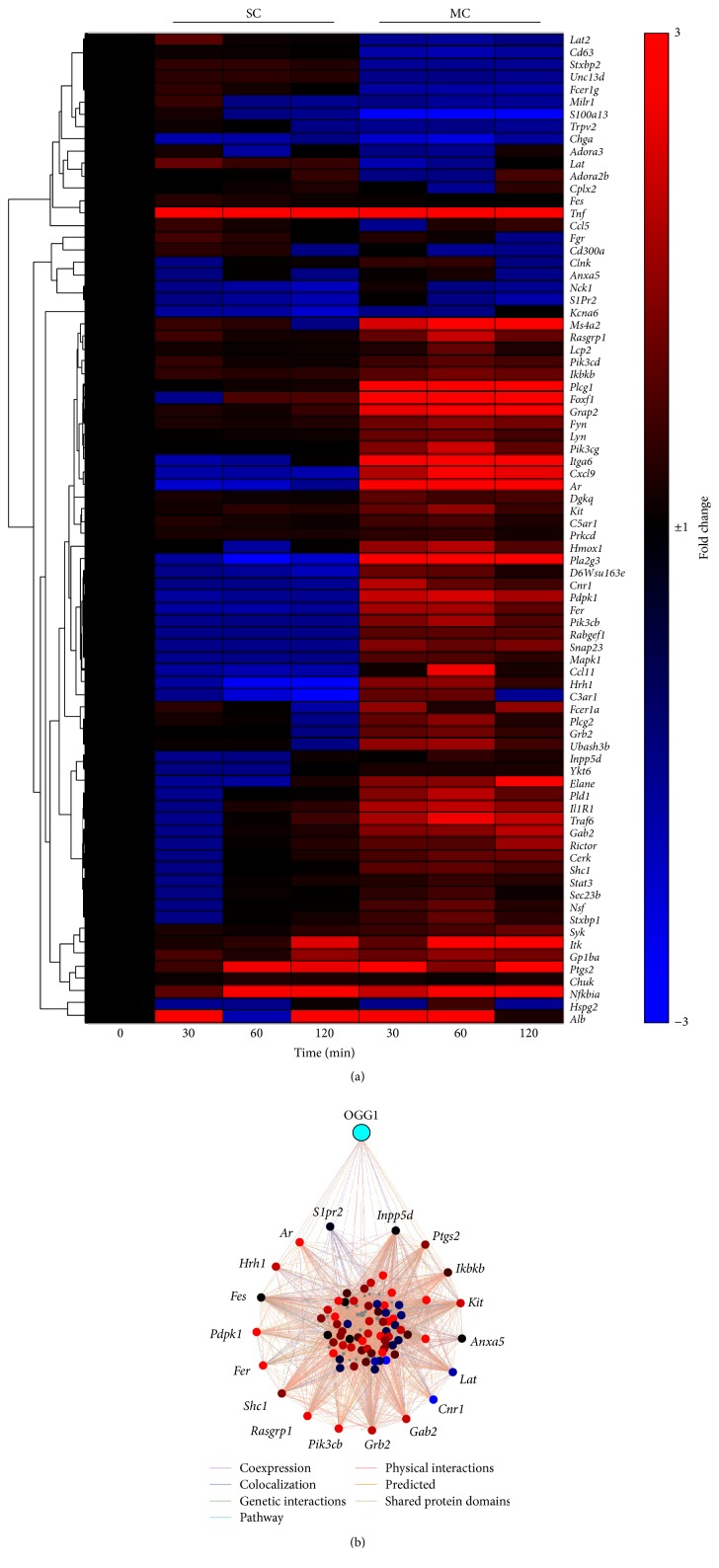
OGG1-BER induced gene expression associated to airway mast cell degranulation. Mouse lungs received a single challenge (SC) or multiple challenges (MC) with 8-oxoG. Lungs were collected at 30 and 60 min after challenge and analyzed at whole-transcriptome level by RNA-Seq. (a) Hierarchically clustered heat map was generated using GENE-E online software (Broad Institute, http://www.broadinstitute.org/) considering transcript levels (fold-change). Red represents upregulation and blue represents downregulation. (b) Enrichment interaction network of the known and predicted interactions among the set associated with mast cell degranulation and OGG1. Peripheral nodes represent genes with a direct interaction with OGG1. The modified network was generated with GeneMania online database (http://www.genemania.org/).

Accordingly, gene expression induced by the most abundant product of DNA oxidative damage/repair (8-oxoG) was associated with mast cell degranulation [77]. To determine the validity of this association, we referenced the human genes database GeneCards® online v 4.0 (http://www.genecards.org/) for a list of 80 genes associated to mast cell degranulation and compared it to our RNA-Seq datasets. A hierarchically clustered heat map (Figure 2(a)) depicts the expression profile of those genes across the datasets from mouse lungs after a SC or a MC with 8-oxoG. This heat map shows an important number of upregulated genes associated with mast cell degranulation in the MC group. The expression at mRNA levels (fold-change) of top upregulated genes is shown in Table 1. Among the highest expressed genes in this group, integrin alpha 6 (*Itga6*) was increased by nearly 30-fold. Interestingly, studies showed increased expression levels of this gene in airway smooth muscle cells derived from individuals with fatal asthma [78]. In other studies, forkhead box F1 (*Foxf1*), a transcriptional factor expressed in endothelial and smooth muscle cells in the lungs, was associated with interstitial pneumonia [79]. This gene showed a nearly 20-fold increase. The product of* Foxf1* also negatively regulates the expression of mast cell tryptase and the expression of chemoattractants such as CXCL12 which is necessary for mast cell migration [80]. These features make* Foxf1* an important factor in the pathogenesis of lung inflammatory responses, and a potentially important factor in EIA. Furthermore, phospholipase C, Gamma 1 (*Plcg1*) transcript level was increased by over 15-fold; its protein product PLCG1 promotes mast cell activation [81]. The interaction network (Figure 2(b)) depicts the direct and indirect interactions between OGG1 and other differentially expressed genes, with a focus on mast cell modulation. These observations and their further investigation might provide insight on potential targets for development of novel compounds to prevent mast cell degranulation in asthmatics, before performing exercise.

## 6. Airway Hyperresponsiveness

One of the most common features of asthma is airway hyperresponsiveness (AHR) which is defined as an increased sensitivity of the airways to constrictor agonists. According to the World Allergy Organization (WAO), EIA represents a major problem among elite athletes, not just through interfering with their performance, but also through representing a potentially significant health risk, as well as a psychological barrier or disincentive toward pushing the limits of performance.

**Figure 3:**
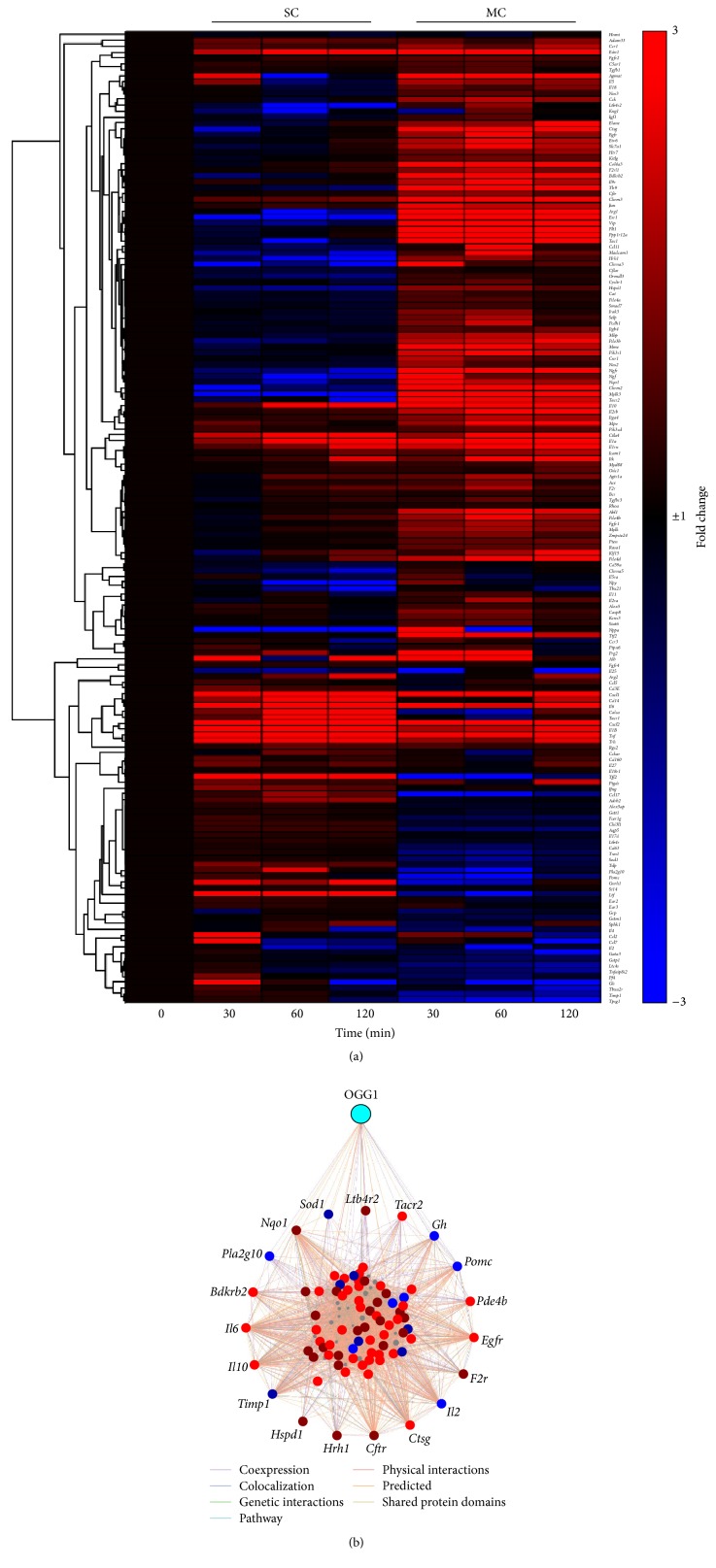
OGG1-BER induced gene expression associated with AHR. Mouse lungs received a single challenge (SC) or multiple challenges (MC) of 8-oxoG and were processed as previously described above. (a) Hierarchically clustered heat map was generated using GENE-E online software (Broad Institute, http://www.broadinstitute.org/) considering transcript levels (fold-change). Red represents upregulation and blue represents downregulation. (b) Enrichment network of the known and predicted interactions among the set of genes associated to airway hyperresponsiveness and OGG1. Peripheral nodes represent genes with a direct interaction with OGG1. The modified network was generated with GeneMania online database (http://www.genemania.org/).

AHR involves various cytokines/chemokines as well as many other mediators, which are under genetic control, and may have an association with the OGG1-BER pathway. To determine the association of OGG1-BER signaling in AHR and the potential link to EIA, we searched in GeneCards database for a list of genes associated with AHR. GeneCards generated a list of 165 genes which was compared to our RNA-Seq datasets from 8-oxoG challenged lungs. A heat map (Figure 3(a)) shows an increased number of upregulated genes in the MC compared to the SC group. This suggested that upregulation of AHR-associated genes might be due to continuous or repeated oxidative damage to the airways, rather than a single insult. The interaction network depicts the multiple interactions between OGG1 and AHR-associated genes (Figure 3(b)). Among the genes with the highest expression levels, interleukin 10 (*Il10*) showed over 30-fold increase at 60 min in the MC group and over 6-fold increase at 60 min in the SC group. This cytokine has anti-inflammatory properties and its upregulation is commonly observed after or during inflammation, as a counter-regulatory off-switch that promotes the normal resolution of inflammation [82]. However, asthmatics have been shown to be deficient in airway IL-10 expression [83], for reasons that continue to be unexplained, but this deficiency may be important for the postulate that OGG1-BER is a critical factor in EIA. In support of this idea, protein levels of IL-10 have been observed to be increased in an exercise animal model in which LPS-induced lung neutrophilia was decreased after exercise only in* Il10* ^+/+^ but not in* Il10* ^−/−^ mice [84]. Thus, it can be theorized that exercise may be beneficial in promoting the IL-10 response in normal individuals. However, in asthmatics and specifically in the case of EIA, exercise may induce an inflammatory oxidative stress that cannot be countered by IL-10, due to deficiency in its production. In the case of the postulated OGG1-BER pathway in EIA, this would create a scenario in which the effects of mast cell and/or neutrophil degranulation would be unchecked by the anti-inflammatory effects of IL-10 [82], resulting in the enhancement of AHR and the diminution of exercise performance.

Furthermore, the expression levels of other genes like* Cxcl1*,* Cxcl2,* and* Tnf* were notably increased. This may be important, because the chemokines CXCL1 and CXCL2 are potent neutrophil chemoattractants which play a role in the pathogenesis and progression of asthma that could be associated to EIA. Moreover, the upregulation of* Tnf* gene expression is again consistent with the upregulation of the* il10* gene, as its protein product TNF-*α* is a major driver of IL-10 release from leukocytes [82] that are either drawn in with inflammation, or perpetually resident, within the airway. In this case, it may be important to consider that IL-10 is self-regulating, in that it normally feeds back to shut down both its own release and that of TNF-*α* [82]; but, with the IL-10 deficiency of asthma, there would be no brake on TNF-*α*, which has its own important proinflammatory and pro-AHR effects [85], which could drive EIA.  Table 2 shows a complete list of the AHR-associated genes and their expression values.

## 7. The Potential Role of OGG1-BER Signaling in EIA

Physical exercise renders physiological stress to the body including oxidative stress [86, 87] and potentially affects the activation of transcription factors such as NF-*κ*B and activator protein-1 (AP1) which stimulates the expression of cytokines and chemokines, to promote physiological responses that may be linked to EIA [88]. Physical factors, such as temperature, can also exacerbate the outcomes in EIA, through altering the redox state of the airways. For example, it has been documented that exposure to cold temperatures during exercise increases oxidative stress levels, as determined by increased plasma ROS metabolites and lower levels of antioxidants [89]. At the cellular level, it has been observed that the increased levels of ROS in the airway epithelium can stimulate the production of leukotrienes and other inflammatory mediators, playing a pivotal role in lung inflammation [90]. Although it has long been known that ROS are linked to proinflammatory gene expression, the precise mechanisms are still not completely understood. As outlined above, recent studies suggest that oxidative stress can play an important role in cell signaling through a DNA-BER initiated signaling pathway. Of note, these studies showed that the repair product of oxidatively damaged DNA by OGG1 forms a complex with OGG1 in the cytosol and activates small GTPases [59, 61, 62] and downstream signaling molecules involved in both the NF-*κ*B signaling pathway and the expression of innate immune response genes [18].

**Figure 4:**
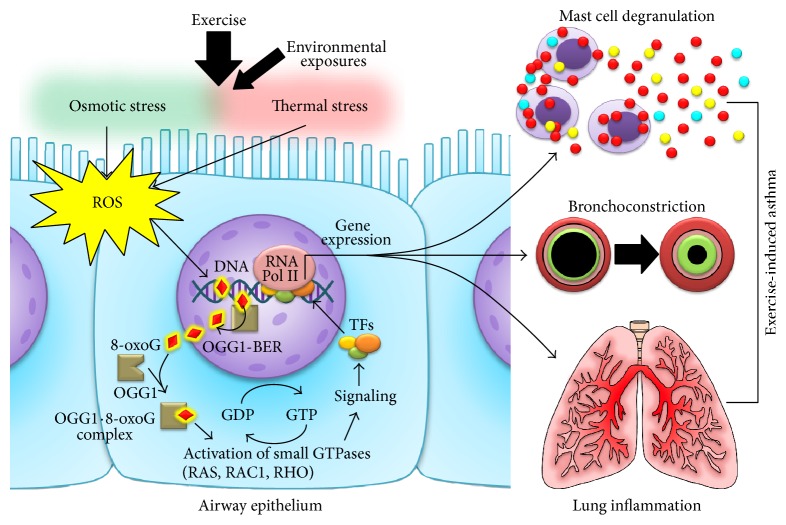
Proposed role for OGG1-BER in asthma and EIA-related gene expression. The large inhaled air volumes during physical activity decrease the temperature and water content of the airway epithelium lining fluid, leading to supraphysiological ROS production by epithelial cells, which can be exacerbated by exposures to environmental pollutants. ROS-induced 8-oxoG lesions in the DNA are repaired by OGG1-BER. In the cytosol, 8-oxoG complexes with OGG1 and acts as a guanine nucleotide exchange factor (GEF), activating small GTPases and initiating a signaling cascade that leads to the translocation of transcription factors (TFs) initiating the transcription of genes associated to AHR, mast cell degranulation, and bronchoconstriction.

Based on the data and previous findings outlined above, we propose a model (Figure 4) that represents the potential role of OGG1-BER signaling in EIA. Within this schema, vigorous exercise results in osmotic and thermal changes in the airways leading to oxidative stress. This condition can be exacerbated by the presence of environmental toxicants that can act as prooxidants, such as ozone and sulfur dioxide [91]. Consequently, DNA in the airway cells is oxidized by ROS and the subsequent 8-oxoG lesions are repaired by OGG1-BER, increasing the levels of cytoplasmic free 8-oxoG leading to the formation of OGG1·8-oxoG complexes. These complexes activate small GTPases such as RAS, RHO, and RAC, inducing downstream signaling which activates transcription factors leading to EIA-related gene expression. The proinflammatory mediators released by airway epithelial and resident cells, such as dendritic cells and macrophages, can result in the typical EIA symptoms including bronchoconstriction, airway hyperresponsiveness, shortness of breath, wheezing, and coughing. It is important to note that IL-10 is conspicuous by its absence in this scheme of OGG1-BER-associated effects on EIA, because of its deficiency in asthmatics [82, 83]. It is likely to be present in nonasthmatics, in amounts necessary to counter the release and effects of proinflammatory mediators. This may also be why some relief from EIA may be obtained with asthma controller treatment therapies such as inhaled corticosteroids [92, 93], which are known to upregulate IL-10 production, in addition to other important anti-inflammatory effects [94].

## 8. Conclusions 

EIA is an acute condition characterized by reversible bronchoconstriction, wheezing, chest tightening, and shortness of breath, during or after exercise. Despite the high prevalence of these potentially serious symptoms in EIA, there is limited information on the molecular pathogenesis. It is mostly agreed that osmotic/thermal changes and environmental pollutants increase levels of oxidative stress; however, the molecular mechanisms by which reactive species are implicated in the pathogenesis of EIA are not fully known. Thus, this review presents a general overview on the pathogenesis of EIA and discusses its potential link to the present knowledge of cell signaling initiated by repair of oxidatively damaged DNA.

However, the genetic basis of EIA has not been completely elucidated. Therefore, we proposed a novel hypothesis (Figure 4), in which cell signaling induced by the repair of oxidatively damaged DNA by OGG1 during the base excision repair pathway plays a role in the regulation of gene expression associated with common symptoms of EIA. This hypothesis is supported by previous studies from other research groups and from our own research group, showing the involvement of OGG1-BER in gene expression related to airway inflammation and other asthma features [18–20, 56, 95]. It is currently acknowledged that osmotic/thermal changes and environmental pollutants increase levels of oxidative stress; however, the understanding of the molecular mechanisms by which reactive species are implicated in the pathogenesis of EIA require further studies. It is evident from the present analysis that DNA damage/repair could be involved in EIA pathogenesis. Further investigation of DNA damage/induced signaling pathway may result in novel pharmacological targets to prevent or treat EIA.

## Figures and Tables

**Table 1:** OGG1-BER induced expression of genes involved in mast cell degranulation.

Symbol	Name	GenBank/RefSeq ID	SC (fold)	MC (fold)
*Itga6*	Integrin alpha 6	NM_008397	−1.28	29.12
*Foxf1*	Forkhead box F1	NM_010426	1.63	18.98
*Plcg1*	Phospholipase C, gamma 1	NM_021280	1.15	17.11
*Alb*	Albumin	NM_009654	−1.74	15.28
*Pla2g3*	Phospholipase A2, group III	NM_172791	−7.06	9.71
*Ar*	Androgen receptor	NM_013476	−2.16	9.58
*Cxcl9*	Chemokine (C-X-C motif) ligand 9	NM_008599	−1.51	9.09
*Ms4a2*	Membrane-spanning 4-domains, subfamily A, member 2	NM_013516	1.33	8.38
*Tnf*	Tumor necrosis factor	NM_013693	16.65	7.13
*Nfkbia*	NFKLP^a^ gene enhancer in B cells inhibitor, alpha	NM_010907	4.73	4.41
*Grap2*	GRB2^b^-related adaptor protein 2	NM_010815	1.18	3.53
*Itk*	Interleukin 2 inducible T cell kinase	NM_010583	1.38	3.34
*Traf6*	Tumor necrosis factor receptor-associated factor 6	NM_009424	1.09	2.94
*Ccl11*	Chemokine (C-C motif) ligand 11	NM_011330	−1.71	2.93
*Pdpk1*	3-phosphoinositide dependent protein kinase 1	NM_011062	−1.22	2.73
*Pik3cg*	Phosphoinositide-3-kinase, catalytic, gamma polypeptide	NM_020272	1.02	2.67
*Rasgrp1*	RAS^c^ guanyl releasing protein 1	NM_011246	1.21	2.62
*Il1r1*	Interleukin 1 receptor, type I	NM_008362	1.24	2.55
*Pld1*	Phospholipase D1	NM_008875	1.02	2.49
*Hmox1*	Heme oxygenase 1	NM_010442	−1.43	2.46
*Pik3cb*	Phosphatidylinositol 3-kinase, catalytic, beta polypeptide	NM_029094	−1.02	2.34
*Fer*	Fer (fms/fps related) protein kinase	NM_008000	−1.60	2.33
*Ubash3b*	Ubiquitin associated and SH3 domain containing, B	NM_176860	1.12	2.25
*Kit*	Kit oncogene	NM_021099	1.39	2.20
*Gp1ba*	Glycoprotein 1b, alpha polypeptide	NM_010326	1.28	2.16
*Hrh1*	Histamine receptor H1	NM_008285	−2.85	2.15
*Fyn*	Fyn proto-oncogene	NM_008054	1.21	2.14
*Plcg2*	Phospholipase C, gamma 2	NM_172285	1.08	2.12
*Elane*	Elastase, neutrophil expressed	NM_015779	−1.43	2.09
*Gab2*	Growth factor receptor bound protein 2-associated protein 2	NM_010248	1.15	2.09

SC, single 8-oxoG challenge; MC, multiple 8-oxoG challenge; NFKLP^a^, nuclear factor of kappa light polypeptide; GRB2^b^, growth factor receptor-bound protein 2. RAS^c^, rat sarcoma. The table shows the top ranked genes (MC, 60 min) by expression level (≥2-fold).

**Table 2:** OGG1-BER induced expression of genes associated to AHR.

Symbol	Name	GenBank/RefSeq ID	SC (fold)	MC (fold)
*Cxcl2*	Chemokine (C-X-C motif) ligand 2	NM_009140	192.95	48.55
*Bdkrb2*	Bradykinin receptor, beta 2	NM_009747	−1.35	45.16
*Il10*	Interleukin 10	NM_010548	5.84	33.51
*Tlr9*	Toll-like receptor 9	NM_031178	−1.54	25.97
*Arg1*	Arginase, liver	NM_007482	−5.00	17.71
*Cxcl1*	Chemokine (C-X-C motif) ligand 1	NM_008176	54.45	15.94
*Esr1*	Estrogen receptor 1 (alpha)	NM_007956	−5.19	15.56
*Alb*	Albumin	NM_009654	−1.74	15.28
*Chrm3*	Cholinergic receptor, muscarinic 3, cardiac	NM_033269	1.66	9.99
*Flt1*	Fms-like tyrosine kinase 1	NM_010228	−1.13	9.06
*Chrm2*	Cholinergic receptor, muscarinic 2, cardiac	NM_203491	−1.79	8.73
*Vip*	Vasoactive intestinal polypeptide	NM_011702	−2.10	7.26
*Tnf*	Tumor necrosis factor	NM_013693	16.65	7.13
*Col4a3*	Collagen, type IV, alpha 3	NM_007734	1.15	6.74
*Tac1*	Tachykinin 1	NM_009311	−4.28	6.70
*Mylk3*	Myosin light chain kinase 3	NM_175441	−4.08	6.35
*Ppp1r12a*	Protein phosphatase 1, regulatory (inhib) subunit 12A	NM_027892	−1.08	6.06
*Ctla4*	Cytotoxic T-lymphocyte-associated protein 4	NM_009843	7.08	5.82
*Madcam1*	Mucosal vascular addressin cell adhesion molecule 1	NM_013591	−1.63	5.66
*Il1a*	Interleukin 1 alpha	NM_010554	4.17	5.55
*Agmat*	Agmatine ureohydrolase (agmatinase)	NM_001081408	−4.28	5.03
*Il1Rn*	Interleukin 1 receptor antagonist	NM_031167	1.92	4.10
*Il2Rb*	Interleukin 2 receptor, beta chain	NM_008368	1.31	4.04
*Il9R*	Interleukin 9 receptor	NM_008374	1.08	3.96
*Edn1*	Endothelin 1	NM_010104	3.10	3.81
*Prg2*	Proteoglycan 2, bone marrow	NM_008920	2.21	3.72
*Mme*	Membrane metallo-endopeptidase	NM_008604	−1.07	3.66
*Ctsg*	Cathepsin G	NM_007800	−1.14	3.35
*Itk*	IL2 inducible T cell kinase	NM_010583	1.38	3.34
*Pik3r1*	PI3K^a^, regulatory subunit, polypeptide 1 (p85 alpha)	NM_001024955	−1.18	3.24
*Mpo*	Myeloperoxidase	NM_010824	1.36	3.24
*Ngfr*	Nerve growth factor receptor	NM_033217	−1.88	3.20
*Slc7a1*	Solute carrier family 7, member 1	NM_007513	−1.28	3.19
*Pde4d*	Phosphodiesterase 4D, cAMP specific	NM_011056	1.08	3.18
*Pde4b*	Phosphodiesterase 4B, cAMP specific	NM_019840	1.39	3.17
*Egfr*	Epidermal growth factor receptor	NM_007912	−1.16	3.05
*Abl1*	C-abl oncogene 1, non-receptor tyrosine kinase	NM_001112703	1.11	3.01
*Etv6*	Ets variant 6	NM_007961	−1.05	2.98
*Tacr2*	Tachykinin receptor 2	NM_009314	−1.07	2.93
*Ccl11*	Chemokine (C-C motif) ligand 11	NM_011330	−1.71	2.93
*Ttf2*	Transcription termination factor, RNA polymerase II	NM_001013026	1.06	2.92
*Il6*	Interleukin 6	NM_031168	10.44	2.75
*Pde3b*	Phosphodiesterase 3B, cGMP-inhibited	NM_011055	−1.79	2.68
*Pcdh1*	Protocadherin 1	NM_029357	−1.12	2.66
*Jun*	Jun proto-oncogene	NM_010591	1.38	2.55
*Nqo1*	NAD(P)H^b^ dehydrogenase, quinone 1	NM_008706	−2.58	2.54
*Il1b*	Interleukin 1 beta	NM_008361	4.15	2.52
*Cck*	Cholecystokinin	NM_031161	−1.43	2.51
*F2rl1*	Coagulation factor II (thrombin) receptor-like 1	NM_007974	1.09	2.51
*Mbp*	Myelin basic protein	NM_010777	−1.40	2.48
*Itga4*	Integrin alpha 4	NM_010576	1.26	2.46
*F2r*	Coagulation factor II (thrombin) receptor	NM_010169	1.46	2.39
*Selp*	Selectin, platelet	NM_011347	−1.31	2.39
*Il2ra*	Interleukin 2 receptor, alpha chain	NM_008367	1.06	2.34
*Agtr1a*	Angiotensin II receptor, type 1a	NM_177322	1.72	2.33
*Fgfr1*	Fibroblast growth factor receptor 1	NM_010206	1.02	2.32
*Mylk*	Myosin, light polypeptide kinase	NM_139300	1.24	2.30
*Klf15*	Kruppel-like factor 15	NM_023184	1.41	2.30
*Ltb4r2*	Leukotriene B4 receptor 2	NM_020490	−7.37	2.15
*Hrh1*	Histamine receptor H1	NM_008285	−2.85	2.15
*Il5*	Interleukin 5	NM_010558	−1.87	2.14
*Icam1*	Intercellular adhesion molecule 1	NM_010493	1.30	2.12
*Irak3*	Interleukin-1 receptor-associated kinase 3	NM_028679	−1.19	2.12
*Cftr*	Cystic fibrosis transmembrane conductance regulator	NM_021050	1.09	2.11
*Elane*	Elastase, neutrophil expressed	NM_015779	−1.43	2.09
*Hspd1*	Heat shock protein 1 (chaperonin)	NM_010477	−2.07	2.06

SC, single 8-oxoG challenge; MC, multiple 8-oxoG challenge; PI3K^a^, phosphatidylinositol 3-kinase; NAD(P)H^b^, dihydronicotinamide-adenine dinucleotide phosphate. The table shows the top ranked genes (MC, 60 min) by expression level (≥2-fold).

## Abbreviations

8-oxoG: 8-Oxo-7,8-dihydroguanine
AHR: Airway hyperresponsiveness
BER: Base excision repair
CysLTs: Cysteinyl leukotrienes
EIA: Exercise-induced asthma
EIB: Exercise-induced bronchoconstriction
FapyG: 2,6-Diamino-4-hydroxy-5-formamidopyrimidine
GEF: Guanine nucleotide exchange factor
H-Ras: Harvey rat sarcoma viral oncogene homolog
IL: Interleukin
K-Ras: Kirsten rat sarcoma viral oncogene homolog
LPS: Lipopolysaccharides
N-Ras: Neuroblastoma RAS viral oncogene homolog
MC: Multiple challenges
OGG1: 8-Oxoguanine DNA glycosylase-1
PI3K: Phosphatidyl inositol 3 kinase
RAC1: Ras-related C3 botulinum toxin substrate 1 GTPase
RHO: Ras homology (Rho) family of small GTPases
ROS: Reactive oxygen species
SC: Single challenge.

## References

[1] WHO (2007). *Global Surveillance, Prevention and Control of Chronic Respiratory Diseases*.

[2] CDC.gov (2015). *Centers for Disease Control and Prevention—Fast Stats*.

[3] McFadden E. R., Gilbert I. A. (1994). Exercise-induced asthma. *The New England Journal of Medicine*.

[4] Dickinson J. W., Whyte G. P., McConnell A. K., Nevill A. M., Harries M. G. (2006). Mid-expiratory flow versus FEV_1_ measurements in the diagnosis of exercise induced asthma in elite athletes. *Thorax*.

[5] Parsons J. P., Craig T. J., Stoloff S. W. (2011). Impact of exercise-related respiratory symptoms in adults with asthma: exercise-induced Bronchospasm Landmark National survey. *Allergy and Asthma Proceedings*.

[6] Carlsen K.-H., Carlsen K. C. L. (2002). Exercise-induced asthma. *Paediatric Respiratory Reviews*.

[7] Randolph C. (2009). An update on exercise-induced bronchoconstriction with and without asthma. *Current Allergy and Asthma Reports*.

[8] Wanrooij V. H. M., Willeboordse M., Dompeling E., van de Kant K. D. G. (2014). Exercise training in children with asthma: a systematic review. *British Journal of Sports Medicine*.

[9] Sun W., Wang Z., Cao J., Cui H., Ma Z. (2016). Cold stress increases reactive oxygen species formation via TRPA1 activation in A549 cells. *Cell Stress and Chaperones*.

[10] Brocker C., Thompson D. C., Vasiliou V. (2012). The role of hyperosmotic stress in inflammation and disease. *Biomolecular Concepts*.

[11] Krafczyk M. A., Asplund C. A. (2011). Exercise-induced bronchoconstriction: diagnosis and management. *American Family Physician*.

[12] Hallstrand T. S., Altemeier W. A., Aitken M. L., Henderson W. R. (2013). Role of cells and mediators in exercise-induced bronchoconstriction. *Immunology and Allergy Clinics of North America*.

[13] Lee J.-U., Kim J. D., Park C.-S. (2015). Gene-environment interactions in asthma: genetic and epigenetic effects. *Yonsei Medical Journal*.

[14] Araneda O. F., Carbonell T., Tuesta M. (2016). Update on the mechanisms of pulmonary inflammation and oxidative imbalance induced by exercise. *Oxidative Medicine and Cellular Longevity*.

[15] Hallstrand T. S. (2012). New insights into pathogenesis of exercise-induced bronchoconstriction. *Current Opinion in Allergy & Clinical Immunology*.

[16] Barnes P. J. (1990). Reactive oxygen species and airway inflammation. *Free Radical Biology and Medicine*.

[17] Tecklenburg S. L., Mickleborough T. D., Fly A. D., Bai Y., Stager J. M. (2007). Ascorbic acid supplementation attenuates exercise-induced bronchoconstriction in patients with asthma. *Respiratory Medicine*.

[18] Aguilera-Aguirre L., Bacsi A., Radak Z. (2014). Innate inflammation induced by the 8-oxoguanine DNA glycosylase-1-KRAS-NF-*κ*B pathway. *The Journal of Immunology*.

[19] Aguilera-Aguirre L., Hosoki K., Bacsi A. (2015). Whole transcriptome analysis reveals an 8-oxoguanine DNA glycosylase-1-driven DNA repair-dependent gene expression linked to essential biological processes. *Free Radical Biology and Medicine*.

[20] Aguilera-Aguirre L., Hosoki K., Bacsi A. (2015). Whole transcriptome analysis reveals a role for OGG1-initiated DNA repair signaling in airway remodeling. *Free Radical Biology and Medicine*.

[21] Ba X., Aguilera-Aguirre L., Sur S., Boldogh I. (2015). 8-Oxoguanine DNA glycosylase-1-driven DNA base excision repair: role in asthma pathogenesis. *Current Opinion in Allergy & Clinical Immunology*.

[22] Hallstrand T. S., Wurfel M. M., Lai Y. (2010). Transglutaminase 2, a novel regulator of eicosanoid production in asthma revealed by genome-wide expression profiling of distinct asthma phenotypes. *PLoS ONE*.

[23] Sahiner U. M., Birben E., Erzurum S., Sackesen C., Kalayci O. (2011). Oxidative stress in asthma. *World Allergy Organization Journal*.

[24] Hosoki K., Kainuma K., Toda M. (2014). Montelukast suppresses epithelial to mesenchymal transition of bronchial epithelial cells induced by eosinophils. *Biochemical and Biophysical Research Communications*.

[25] Kirkham P., Rahman I. (2006). Oxidative stress in asthma and COPD: antioxidants as a therapeutic strategy. *Pharmacology and Therapeutics*.

[26] Montuschi P., Barnes P. J., Roberts L. J. (2004). Isoprostanes: markers and mediators of oxidative stress. *The FASEB Journal*.

[27] Okazawa A., Kawikova I., Cui Z.-H., Skoogh B.-E., Lötvall J. (1997). 8-Epi-PGF2alpha induces airflow obstruction and airway plasma exudation in vivo. *American Journal of Respiratory and Critical Care Medicine*.

[28] Montuschi P., Corradi M., Ciabattoni G., Nightingale J., Kharitonov S. A., Barnes P. J. (1999). Increased 8-isoprostane, a marker of oxidative stress, in exhaled condensate of asthma patients. *American Journal of Respiratory and Critical Care Medicine*.

[29] Barreto M., Villa M. P., Olita C., Martella S., Ciabattoni G., Montuschi P. (2009). 8-Isoprostane in exhaled breath condensate and exercise-induced bronchoconstriction in asthmatic children and adolescents. *Chest*.

[30] Lundeen K. A., Sun B., Karlsson L., Fourie A. M. (2006). Leukotriene B4 receptors BLT1 and BLT2: expression and function in human and murine mast cells. *Journal of Immunology*.

[31] Sala A., Zarini S., Bolla M. (1998). Leukotrienes: lipid bioeffectors of inflammatory reactions. *Biochemistry*.

[32] Torres-Atencio I., Ainsua-Enrich E., de Mora F., Picado C., Martín M. (2014). Prostaglandin E_2_ prevents hyperosmolar-induced human mast cell activation through prostanoid receptors EP_2_ and EP_4_. *PLoS ONE*.

[33] Hallstrand T. S., Moody M. W., Wurfel M. M., Schwartz L. B., Henderson W. R., Aitken M. L. (2005). Inflammatory basis of exercise-induced bronchoconstriction. *American Journal of Respiratory and Critical Care Medicine*.

[34] Capra V., Ambrosio M., Riccioni G., Rovati G. E. (2006). Cysteinyl-leukotriene receptor antagonists: present situation and future opportunities. *Current Medicinal Chemistry*.

[35] Philteos G. S., Davis B. E., Cockcroft D. W., Marciniuk D. D. (2005). Role of leukotriene receptor antagonists in the treatment of exercise-induced bronchoconstriction: a review. *Allergy, Asthma & Clinical Immunology*.

[36] Margolin Y., Cloutier J.-F., Shafirovich V., Geacintov N. E., Dedon P. C. (2006). Paradoxical hotspots for guanine oxidation by a chemical mediator of inflammation. *Nature Chemical Biology*.

[37] Steenken S., Jovanovic S. V. (1997). How easily oxidizable is DNA? One-electron reduction potentials of adenosine and guanosine radicals in aqueous solution. *Journal of the American Chemical Society*.

[38] Hickerson R. P., Prat F., Muller J. G., Foote C. S., Burrows C. J. (1999). Sequence and stacking dependence of 8-oxoguanine oxidation: comparison of one-electron vs singlet oxygen mechanisms. *Journal of the American Chemical Society*.

[39] Burrows C. J., Muller J. G. (1998). Oxidative nucleobase modifications leading to strand scission. *Chemical Reviews*.

[40] Kino K., Sugiyama H. (2000). GC → CG transversion mutation might be caused by 8-oxoguanine oxidation product. *Nucleic Acids Symposium Series*.

[41] Dizdaroglu M. (1985). Formation of 8-hydroxyguanine moiety in deoxyribonucleic acid on .gamma.-irradiation in aqueous solution. *Biochemistry*.

[42] Izumi T., Wiederhold L. R., Roy G. (2003). Mammalian DNA base excision repair proteins: their interactions and role in repair of oxidative DNA damage. *Toxicology*.

[43] Dizdaroglu M., Kirkali G., Jaruga P. (2008). Formamidopyrimidines in DNA: mechanisms of formation, repair, and biological effects. *Free Radical Biology and Medicine*.

[44] Lindahl T., Barnes D. E. (2000). Repair of endogenous DNA damage. *Cold Spring Harbor Symposia on Quantitative Biology*.

[45] Nehls P., Seiler F., Rehn B., Greferath R., Bruch J. (1997). Formation and persistence of 8-oxoguanine in rat lung cells as an important determinant for tumor formation following particle exposure. *Environmental Health Perspectives*.

[46] Loft S., Poulsen H. E., Vistisen K., Knudsen L. E. (1999). Increased urinary excretion of 8-oxo-2′-deoxyguanosine, a biomarker of oxidative DNA damage, in urban bus drivers. *Mutation Research/Genetic Toxicology and Environmental Mutagenesis*.

[47] Barbato D. L., Tomei G., Tomei F., Sancini A. (2010). Traffic air pollution and oxidatively generated DNA damage: can urinary 8-oxo-7,8-dihydro-2-deoxiguanosine be considered a good biomarker? A meta-analysis. *Biomarkers*.

[48] Hasbal C., Aksu B. Y., Himmetoglu S. (2010). DNA damage and glutathione level in children with asthma bronchiale: effect of antiasthmatic therapy. *Pediatric Allergy and Immunology*.

[49] Al-Afaleg N. O., Al-Senaidy A., El-Ansary A. (2011). Oxidative stress and antioxidant status in Saudi asthmatic patients. *Clinical Biochemistry*.

[50] Proklou A., Soulitzis N., Neofytou E. (2013). Granule cytotoxic activity and oxidative DNA damage in smoking and nonsmoking patients with asthma. *Chest*.

[51] Deslee G., Woods J. C., Moore C. (2009). Oxidative damage to nucleic acids in severe emphysema. *Chest*.

[52] Igishi T., Hitsuda Y., Kato K. (2003). Elevated urinary 8-hydroxydeoxyguanosine, a biomarker of oxidative stress, and lack of association with antioxidant vitamins in chronic obstructive pulmonary disease. *Respirology*.

[53] Caramori G., Adcock I. M., Casolari P. (2011). Unbalanced oxidant-induced DNA damage and repair in COPD: a link towards lung cancer. *Thorax*.

[54] Mabley J. G., Pacher P., Deb A., Wallace R., Elder R. H., Szabó C. (2005). Potential role for 8-oxoguanine DNA glycosylase in regulating inflammation. *The FASEB Journal*.

[55] Touati E., Michel V., Thiberge J.-M. (2006). Deficiency in OGG1 protects against inflammation and mutagenic effects associated with H. pylori infection in mouse. *Helicobacter*.

[56] Bacsi A., Aguilera-Aguirre L., Szczesny B. (2013). Down-regulation of 8-oxoguanine DNA glycosylase 1 expression in the airway epithelium ameliorates allergic lung inflammation. *DNA Repair*.

[57] Lai C.-C., Boguski M., Broek D., Powers S. (1993). Influence of guanine nucleotides on complex formation between Ras and CDC25 proteins. *Molecular and Cellular Biology*.

[58] Mosteller R. D., Han J., Broek D. (1994). Identification of residues of the H-Ras protein critical for functional interaction with guanine nucleotide exchange factors. *Molecular and Cellular Biology*.

[59] Boldogh I., Hajas G., Aguilera-Aguirre L. (2012). Activation of Ras signaling pathway by 8-oxoguanine DNA glycosylase bound to its excision product, 8-oxoguanine. *The Journal of Biological Chemistry*.

[60] German P., Szaniszlo P., Hajas G. (2013). Activation of cellular signaling by 8-oxoguanine DNA glycosylase-1-initiated DNA base excision repair. *DNA Repair*.

[61] Hajas G., Bacsi A., Aguilera-Aguirre L. (2013). 8-Oxoguanine DNA glycosylase-1 links DNA repair to cellular signaling via the activation of the small GTPase Rac1. *Free Radical Biology and Medicine*.

[62] Luo J., Hosoki K., Bacsi A. (2014). 8-Oxoguanine DNA glycosylase-1-mediated DNA repair is associated with Rho GTPase activation and *α*-smooth muscle actin polymerization. *Free Radical Biology and Medicine*.

[63] Pandita T. K. (2014). Unraveling the novel function of the DNA repair enzyme 8-oxoguanine-DNA glycosylase in activating key signaling pathways. *Free Radical Biology and Medicine*.

[64] Ba X., Aguilera-Aguirre L., Rashid Q. T. A. N. (2014). The role of 8-oxoguanine DNA glycosylase-1 in inflammation. *International Journal of Molecular Sciences*.

[65] Metcalfe D. D., Baram D., Mekori Y. A. (1997). Mast cells. *Physiological Reviews*.

[66] Burgoyne R. D., Morgan A. (2003). Secretory granule exocytosis. *Physiological Reviews*.

[67] Logan M. R., Odemuyiwa S. O., Moqbel R. (2003). Understanding exocytosis in immune and inflammatory cells: the molecular basis of mediator secretion. *Journal of Allergy and Clinical Immunology*.

[68] Rivera J., Gilfillan A. M. (2006). Molecular regulation of mast cell activation. *The Journal of Allergy and Clinical Immunology*.

[69] Frossi B., De Carli M., Pucillo C. (2004). The mast cell: an antenna of the microenvironment that directs the immune response. *Journal of Leukocyte Biology*.

[70] Nagata M. (2005). Inflammatory cells and oxygen radicals. *Current Drug Targets: Inflammation and Allergy*.

[71] Frossi B., De Carli M., Daniel K. C., Rivera J., Pucillo C. (2003). Oxidative stress stimulates IL-4 and IL-6 production in mast cells by an APE/Ref-1-dependent pathway. *European Journal of Immunology*.

[72] Ohmori H., Komoriya K., Azuma A., Kurozumi S., Oto Y. H. (1979). Xanthine oxidase-induced histamine release from isolated rat peritoneal mast cells: involvement of hydrogen peroxide. *Biochemical Pharmacology*.

[73] Swindle E. J., Hunt J. A., Coleman J. W. (2002). A comparison of reactive oxygen species generation by rat peritoneal macrophages and mast cells using the highly sensitive real-time chemiluminescent probe pholasin: inhibition of antigen-induced mast cell degranulation by macrophage-derived hydrogen peroxide. *The Journal of Immunology*.

[74] Bacsi A., Dharajiya N., Choudhury B. K., Sur S., Boldogh I. (2005). Effect of pollen-mediated oxidative stress on immediate hypersensitivity reactions and late-phase inflammation in allergic conjunctivitis. *Journal of Allergy and Clinical Immunology*.

[75] Bacsi A., Choudhury B. K., Dharajiya N., Sur S., Boldogh I. (2006). Subpollen particles: carriers of allergenic proteins and oxidases. *Journal of Allergy and Clinical Immunology*.

[76] Boldogh I., Bacsi A., Choudhury B. K. (2005). ROS generated by pollen NADPH oxidase provide a signal that augments antigen-induced allergic airway inflammation. *The Journal of Clinical Investigation*.

[77] Chodaczek G., Bacsi A., Dharajiya N., Sur S., Hazra T. K., Boldogh I. (2009). Ragweed pollen-mediated IgE-independent release of biogenic amines from mast cells via induction of mitochondrial dysfunction. *Molecular Immunology*.

[78] Himes B. E., Koziol-White C., Johnson M. (2015). Vitamin D modulates expression of the airway smooth muscle transcriptome in fatal asthma. *PLoS ONE*.

[79] Coon D. R., Roberts D. J., Loscertales M., Kradin R. (2006). Differential epithelial expression of *SHH* and *FOXF1* in usual and nonspecific interstitial pneumonia. *Experimental and Molecular Pathology*.

[80] Kalin T. V., Meliton L., Meliton A. Y., Zhu X., Whitsett J. A., Kalinichenko V. V. (2008). Pulmonary mastocytosis and enhanced lung inflammation in mice heterozygous null for the Foxf1 gene. *American Journal of Respiratory Cell and Molecular Biology*.

[81] Tkaczyk C., Beaven M. A., Brachman S. M., Metcalfe D. D., Gilfillan A. M. (2003). The phospholipase C*γ*1-dependent pathway of Fc*ε*RI-mediated mast cell activation is regulated independently of phosphatidylinositol 3-kinase. *The Journal of Biological Chemistry*.

[82] Ogawa Y., Duru E. A., Ameredes B. T. (2008). Role of IL-10 in the resolution of airway inflammation. *Current Molecular Medicine*.

[83] Borish L., Aarons A., Rumbyrt J., Cvietusa P., Negri J., Wenzel S. (1996). Interleukin-10 regulation in normal subjects and patients with asthma. *The Journal of Allergy and Clinical Immunology*.

[84] Reis Gonçalves C. T., Reis Gonçalves C. G., de Almeida F. M. (2012). Protective effects of aerobic exercise on acute lung injury induced by LPS in mice. *Critical Care*.

[85] Lykouras D., Sampsonas F., Kaparianos A., Karkoulias K., Spiropoulos K. (2008). Role and pharmacogenomics of TNF-*α* in asthma. *Mini-Reviews in Medicinal Chemistry*.

[86] Shah N. R., Iqbal M. B., Barlow A., Bayliss J. (2011). Severe physical exertion, oxidative stress, and acute lung injury. *Clinical Journal of Sport Medicine*.

[87] McAnulty S. R., McAnulty L., Pascoe D. D. (2005). Hyperthermia increases exercise-induced oxidative stress. *International Journal of Sports Medicine*.

[88] Hilberg T. (2007). Etiology of exercise-induced asthma: physical stress-induced transcription. *Current Allergy and Asthma Reports*.

[89] Martarelli D., Cocchioni M., Scuri S., Spataro A., Pompei P. (2011). Cold exposure increases exercise-induced oxidative stress. *Journal of Sports Medicine and Physical Fitness*.

[90] Imanifooladi A. A., Yazdani S., Nourani M. R. (2010). The role of nuclear factor-*κ*B in inflammatory lung disease. *Inflammation & Allergy-Drug Targets*.

[91] Reno A. L., Brooks E. G., Ameredes B. T. (2015). Mechanisms of heightened airway sensitivity and responses to inhaled SO_2_ in asthmatics. *Environmental Health Insights*.

[92] Subbarao P., Duong M., Adelroth E. (2006). Effect of ciclesonide dose and duration of therapy on exercise-induced bronchoconstriction in patients with asthma. *Journal of Allergy and Clinical Immunology*.

[93] Umland S. P., Schleimer R. P., Johnston S. L. (2002). Review of the molecular and cellular mechanisms of action of glucocorticoids for use in asthma. *Pulmonary Pharmacology and Therapeutics*.

[94] John M., Lim S., Seybold J. (1998). Inhaled corticosteroids increase interleukin-10 but reduce macrophage inflammatory protein-1 *α*, granulocyte-macrophage colony-stimulating factor, and interferon- *γ* release from alveolar macrophages in asthma. *American Journal of Respiratory and Critical Care Medicine*.

[95] Li G., Yuan K., Yan C. (2012). 8-oxoguanine-DNA glycosylase 1 deficiency modifies allergic airway inflammation by regulating STAT6 and IL-4 in cells and in mice. *Free Radical Biology and Medicine*.

